# Current clinical nutrition practices in critically ill patients in Latin America: a multinational observational study

**DOI:** 10.1186/s13054-017-1805-z

**Published:** 2017-08-25

**Authors:** Karin Papapietro Vallejo, Carolina Méndez Martínez, Alfredo A. Matos Adames, Vanessa Fuchs-Tarlovsky, Guillermo Carlos Contreras Nogales, Roger Enrique Riofrio Paz, Mario Ignacio Perman, Maria Isabel Toulson Davisson Correia, Dan Linetzky Waitzberg

**Affiliations:** 1 Intensive Care and Nutrition Unit, Hospital Clínico de la Universidad de Chile, Santiago de Chile, Chile; 2 Universidad Militar Nueva Granada, Bogotá, Colombia; 3 Complejo Hospitalario de la Caja del Seguro Social de Panamá, Panama City, Panama; 4 Hospital General de México, Mexico City, Mexico; 5 Hospital Guillermo Kaelin de la Fuente, Villa María del Triunfo, Peru; 6 Hospital Metropolitano, Quito, Ecuador; 7 Adult Intensive Care Unit, Department of Medicine, Italian Hospital of Buenos Aires, Buenos Aires, Argentina; 8 Universidade Federal de Minas Gerais Medical School, Av. Carandaí 246 Apt. 902, Belo Horizonte, 30130-060 Brazil; 9 Department of Gastroenterology, University of São Paulo Medical School, São Paulo, Brazil

**Keywords:** Disease-related malnutrition, Caloric balance, Intensive care, Enteral nutrition, Parenteral nutrition

## Abstract

**Background:**

Malnutrition in critically ill adults in the intensive care unit (ICU) is associated with a significantly elevated risk of mortality. Adequate nutrition therapy is crucial to optimise outcomes. Currently, there is a paucity of such data in Latin America. Our aims were to characterise current clinical nutrition practices in the ICU setting in Latin America and evaluate whether current practices meet caloric and protein requirements in critically ill patients receiving nutrition therapy.

**Methods:**

We conducted a cross-sectional, retrospective, observational study in eight Latin American countries (Argentina, Brazil, Chile, Colombia, Ecuador, Mexico, Panama, and Peru). Eligible patients were critically ill adults hospitalised in the ICU and receiving enteral nutrition (EN) and/or parenteral nutrition (PN) on the Screening Day and the previous day (day −1). Caloric and protein balance on day –1, nutritional status, and prescribed nutrition therapy were recorded. Multivariable logistic regression analysis was performed to identify independent predictors of reaching daily caloric and protein targets.

**Results:**

The analysis included 1053 patients from 116 hospitals. Evaluation of nutritional status showed that 74.1% of patients had suspected/moderate or severe malnutrition according to the Subjective Global Assessment. Prescribed nutrition therapy included EN alone (79.9%), PN alone (9.4%), and EN + PN (10.7%). Caloric intake met >90% of the daily target in 59.7% of patients on day –1; a caloric deficit was present in 40.3%, with a mean (±SD) daily caloric deficit of –688.8 ± 455.2 kcal. Multivariable logistic regression analysis showed that combined administration of EN + PN was associated with a statistically significant increase in the probability of meeting >90% of daily caloric and protein targets compared with EN alone (odds ratio, 1.56; 95% confidence interval, 1.02–2.39; *p* = 0.038).

**Conclusions:**

In the ICU setting in Latin America, malnutrition was highly prevalent and caloric intake failed to meet targeted energy delivery in 40% of critically ill adults receiving nutrition therapy. Supplemental administration of PN was associated with improved energy and protein delivery; however, PN use was low. Collectively, these findings suggest an opportunity for more effective utilisation of supplemental PN in critically ill adults who fail to receive adequate nutrition from EN alone.

**Electronic supplementary material:**

The online version of this article (doi:10.1186/s13054-017-1805-z) contains supplementary material, which is available to authorized users.

## Background

Disease-related malnutrition in hospitalised patients is a highly prevalent but frequently under-recognised condition and a major public health problem [1]. Poor nutritional status is associated with significant clinical and economic consequences, including increased risk of infectious and non-infectious complications, prolonged duration of stay in the hospital and intensive care unit (ICU), more frequent readmission, and increased mortality [2–10]. This is especially true in critically ill patients, as the catabolic state induced by the systemic inflammatory response to critical illness or trauma markedly increases metabolic demands, thereby accelerating the development of malnutrition and further increasing the risk of infectious complications, multi-organ dysfunction, and mortality [11, 12].

Providing adequate nutrition is an integral part of the treatment of critically ill patients [11, 12]. Evidence suggests it can attenuate the metabolic response to stress, prevent cellular injury, and promote a favourable immune response [11]. Studies in medical and surgical intensive care populations have demonstrated that adequate nutrition therapy is associated with a decrease in infectious morbidity [13, 14], length of hospital stay [13–15], and mortality [15, 16]. Current clinical practice guidelines for the nutritional management of critically ill patients differ with respect to the use and timing of initiating parenteral nutrition (PN) as well as the optimal daily caloric and protein intake. The European and North American guidelines advocate early enteral nutrition (EN) in critically ill patients who are unable to maintain oral intake [11, 12, 17]. The European Society for Clinical Nutrition and Metabolism (ESPEN) guidelines further recommend early initiation of PN in all patients for whom EN is contraindicated or not tolerated [12], while the American Society for Parenteral and Enteral Nutrition (A.S.P.E.N.) guidelines recommend early use of PN in patients with evidence of malnutrition on admission when EN is not feasible [11, 17]. Both the ESPEN and A.S.P.E.N. guidelines recommend the use of supplemental PN in patients who are unable to meet the targeted energy and protein intake via the enteral route. The ESPEN guidelines recommend initiating supplemental PN in patients who fail to reach the targeted intake by day 3, while the A.S.P.E.N. guidelines indicate supplemental PN should be considered after 7–10 days in patients who are unable to meet >60% of energy and protein requirements [11, 12]. Recently published clinical practice guidelines from the European Society of Intensive Care Medicine (ESICM) advocate the use of early enteral nutrition in the majority of critically ill patients and identify specific clinical circumstances when EN should be delayed; however, the nutritional management of patients for whom EN is insufficient or contraindicated is not specifically addressed [18].

A recent prospective observational study evaluating the nutritional status of 185 critically ill patients admitted to the ICU in a Brazilian hospital reported an overall prevalence of malnutrition of 54.5%. The prevalence was even higher (70.3%) among patients who were hospitalised more than 48 hours before admission to the ICU [19]. Moreover, multivariate logistic regression analysis showed a twofold increase in the risk of readmission to the ICU and an eightfold increase in the risk of death among patients who were malnourished compared with well-nourished patients. These findings underscore the need for the development of evidence-based clinical nutrition practices aimed at the proactive identification of nutritional needs and the optimal nutritional management of critically ill patients. To gain insights that will inform subsequent recommendations, we conducted a multinational observational study (“Screening Day Latin America”) to characterise current clinical nutrition practices in the intensive care setting in Latin America and evaluate the degree to which current practices meet the daily caloric requirements in critically ill patients receiving EN and/or PN.

## Methods

### Study design

**Fig. 1:**
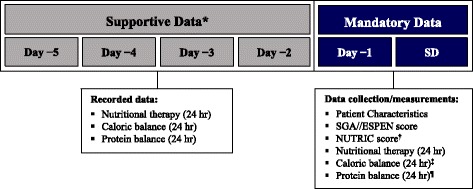
Study design. *Available data from patient records collected for each day of stay in the ICU during the period from day –5 to day –2. ^†^NUTRIC score calculated based on data in the patients’ medical file collected at the time of ICU admission. ^‡^Calculated as the difference between the daily caloric target and the daily calories provided by enteral and/or parenteral nutrition plus other sources of caloric intake. ^¶^Calculated as the difference between the clinician-derived daily protein target and the daily protein intake. *Abbreviations*: *ESPEN* European Society for Clinical Nutrition and Metabolism, *NUTRIC* nutrition risk in the critically ill, *SD* Screening Day, *SGA* Subjective Global Assessment

The Screening Day study was a multinational, cross-sectional, retrospective observational study evaluating clinical nutrition practices in critically ill adults in the intensive care setting in eight Latin American countries (Argentina, Brazil, Chile, Colombia, Ecuador, Mexico, Panama, and Peru). The observation period was defined as the period from the Screening Day (day 0) up to a maximum of 5 days in the ICU before the Screening Day (day −5) (Fig. 1). Demographic and clinical characteristics, nutritional status, nutrition-related risk, type and volume of nutrition therapy, and daily caloric and protein balance during the observation period were assessed by investigators on the Screening Day.

The study protocol was approved by the local ethics committee or institutional review board at each participating institution. Where required by local regulations or ethics committee policy, written informed consent was obtained from each patient or family member before enrollment. The study was funded and organised by Fresenius Kabi Deutschland GmbH, Bad Homberg, Germany. Statistical analysis was performed by IMS Health S.A., Madrid, Spain.

### Study population

Eligible patients were critically ill adults (age ≥18 years) who were hospitalised in the ICU and received EN and/or PN on both the Screening Day (day 0) and the previous day (day −1). For the purpose of eligibility, a critically ill patient was defined as a patient with at least one organ failure; critically ill burn patients and patients admitted to the ICU for surveillance only were excluded from enrollment.

### Measurements and outcomes

The primary study outcome was the daily caloric balance on day −1. The daily caloric balance was calculated as the difference between the daily caloric target and the daily calories provided by EN and/or PN. The daily caloric target was derived by the clinician using one of four methods: calculation using a predictive equation (Harris-Benedict equation), estimation (total daily caloric requirement as estimated by the physician), standard formula (standardised daily target per kilogram actual body weight), or indirect calorimetry. Additionally, a sensitivity analysis of the primary outcome was performed using a standard formula to calculate the daily caloric target for each patient (20 kcal/kg actual body weight on the Screening Day for those on the first 6 days in the ICU and 25 kcal/kg actual body weight on the Screening Day for those in the ICU after day 6). For obese patients (BMI ≥30 kg/m^2^), the caloric target was calculated based on ideal body weight, as determined by height using the formula by Hamwi [20].

Secondary outcomes included the cumulative caloric balance during the observation period (days −5 to −1), daily protein balance on day –1, cumulative protein balance (days –5 to –1), patient nutritional status and nutrition-related risk (day 0), and use of EN and PN during the 5-day observation period. Daily protein balance was calculated as the difference between the clinician-derived daily protein target and the daily protein intake. In contrast to the caloric balance, sensitivity analysis based on a standard equation was not performed, as the defined protein target must be adapted based on certain clinical conditions (e.g. renal and hepatic insufficiency). Cumulative caloric balance and cumulative protein balance were defined as the sum of the mean daily balance for all days from day −5, calculated for each day from day −5 to day −1 (e.g. the cumulative balance on day −3 was calculated as the sum of the mean daily balance on days −5, −4, and −3). Patient nutritional status was determined according to the Subjective Global Assessment (SGA) questionnaire and the ESPEN malnutrition score; nutrition-related risk was determined based on the Nutrition Risk in the Critically Ill (NUTRIC) score (Additional file 1: Appendices A–C) [21–25]. For the latter, the score was calculated based on data in the patients’ medical file collected at the time of ICU admission. Physical exams, patient interviews, and chart reviews were performed by the investigators on the Screening Day. If a patient was unable to participate in the interview, the patient’s relative was interviewed to obtain the requisite information.

Hospital characteristics, including the number of beds in the hospital and ICU, the type and volume of nutrition therapies prescribed, the availability of a nutrition therapy team, and institutional nutrition management policies and practices were recorded by the investigator at each participating institution using an electronic case report form.

### Statistical methods

The analysis population was defined according to the per protocol principle and included all patients with complete data for the protocol-defined mandatory variables and without a protocol violation (e.g. failure to meet all eligibility criteria). Data are summarised descriptively using number and percentage for categorical variables and mean ± standard deviation (SD) as well as median for continuous variables. The primary outcome is presented both as a continuous variable using the mean (SD) difference between the daily caloric target and the daily caloric intake and as a categorical variable using the number and percentage of patients in each of the following categories: meets >90% of daily target and caloric deficit (≤90% of daily target). The primary outcome was also analysed in subpopulations defined according to the following parameters: sex, nutritional status, type of nutritional therapy, reason for admission to the ICU, duration of stay in the ICU before the Screening Day, Acute Physiology and Chronic Health Evaluation (APACHE) II score, and Sequential Organ Failure Assessment (SOFA) score.

Univariable and multivariable logistic regression models were used to explore the relationship between the daily caloric and protein balance on day −1 and selected hospital and patient characteristics. All variables with a *p* value ≤0.20 in the unadjusted univariable analysis were included in the multivariable analysis. Final model specification was based on a backward step-wise elimination of variables with a *p* value >0.05. Results are reported as the adjusted odds ratio (OR) and 95% confidence interval (95% CI) for each variable. All analyses were performed using SAS Enterprise Guide 6.1 running SAS 9.4 (SAS Institute Inc., Cary, NC, USA).

Sample size calculations were based on an estimated prevalence of disease-related malnutrition of 50%, derived from the results of prior multinational epidemiological studies in Latin America [9, 26]. It was determined that a sample size of 2000 patients would provide a confidence interval with a precision of ± 2.2%.

## Results

A total of 1053 patients in 116 hospitals in eight Latin American countries met the criteria for eligibility and consented to participate in the study (Table 1). The majority of participating institutions were academic/university hospitals (94 [81.0%]) providing tertiary care services (93 [80.2%]). A total of 46 (39.7%) hospitals reported having a nutrition therapy team in the ICU; of these, 36 (78.3%) were academic/university hospitals (hospital characteristics are summarised in Additional file 1: Appendix D).

**Table 1:** Study enrollment summary^a^

Country	Hospitals, n	Patients, n
Argentina	16	110
Brazil	13	133
Chile	20	211
Colombia	22	229
Ecuador	14	103
Mexico/Panama	16	129
Peru	15	138
Total	116	1053

^a^See Additional file 1: Appendix F for a complete list of study sites

Patient characteristics are summarised in Table 2. The mean (±SD) age was 58.6 ± 19.0 years and the mean duration of ICU stay on the Screening Day was 27.6 ± 62.2 days. The most common primary reasons for admission to the ICU were respiratory illness (315 [30.0%]), neurologic illness (234 [22.3%]), sepsis (210 [20.0%]), and trauma (102 [9.7%]). Approximately half of the patients (47.3%) were classified as surgical patients. Invasive respiratory support was required in 799 (75.9%) patients. A total of 841 (79.9%) patients received EN only, 113 (10.7%) received both EN and PN, and 99 (9.4%) received PN only.

**Table 2:** Patient characteristics

Characteristic	Total (*N* = 1053)
Age, years	
Mean (SD)	58.6 (19.0)
Median (range)	61.0 (18.0–99.0)
Sex, n (%)	
Male	602 (57.2)
Female	451 (42.8)
Height, cm	
Mean (SD)	163.3 (9.6)
Median (range)	170.0 (135.0–196.0)
Mean weight, kg (SD)	
Screening Day	
Mean (SD)	68.8 (17.6)
Median (range)	66.0 (30.0–195.0)
Admission^a^	
Mean (SD)	71.2 (18.8)
Median (range)	70.0 (30.0–240.0)
Mean BMI, kg/m^2^ (SD)	25.8 (6.1)
Age <70 years	25.6 (6.4)
Age ≥70 years	26.0 (5.6)
BMI, n (%)	
<18.5 kg/m^2^	69 (6.6)
18.5 to <20 kg/m^2^	57 (5.4)
20 to <22 kg/m^2^	131 (12.4)
22 to <30 kg/m^2^	609 (57.8)
≥30 kg/m^2^	187 (17.8)
Type of nutrition, n (%)	
Enteral nutrition only	841 (79.9)
Parenteral nutrition only	99 (9.4)
Both parenteral and enteral nutrition	113 (10.7)
Primary reason for ICU admission, n (%)^b^	
Respiratory	315 (30.0)
Neurologic	234 (22.3)
Sepsis	210 (20.0)
Trauma	102 (9.7)
Abdominal	95 (9.0)
Other	86 (8.2)
Not available	8 (0.8)
Time since ICU admission, days^b^	
Mean (SD)	27.6 (62.2)
Median (range)	10.0 (0–465.0)
Duration of ICU stay on Screening Day, n (%)^b^	
0–5 days	301 (28.7)
6–9 days	192 (18.3)
≥10 days	557 (53.0)
Comorbid conditions, n (%)^c^	
0	137 (14.2)
1–5	795 (82.2)
≥ 5	35 (3.6)
APACHE II score, n (%)	
<15	389 (36.9)
15 to <20	266 (25.3)
20 to <28	282 (26.8)
≥28	116 (11.0)
SOFA score, n (%)	
<6	482 (45.8)
6 to <10	369 (35.0)
≥10	202 (19.2)
Requirement for invasive respiratory support, n (%)	799 (75.9)

*Abbreviations: APACHE* Acute Physiology and Chronic Health Evaluation, *BMI* body mass index, *ICU* intensive care unit, *SD* standard deviation, *SOFA* Sequential Organ Failure Assessment

^a^N = 896

^b^N = 1050

^c^N = 967

### Daily caloric balance

Clinician-derived daily energy requirements were established for 800 (76%) patients using one of the following methods: estimation (44.1%), predictive equation (31.3%), standardised formula (23.4%), or indirect calorimetry (1.3%). In 253 (24%) patients, no clinician-derived energy target was given. In these patients, a daily caloric target of 20 kcal/kg actual body weight (25 kcal/kg for patients with ≥6 days in the ICU) was assigned.

**Fig. 2:**
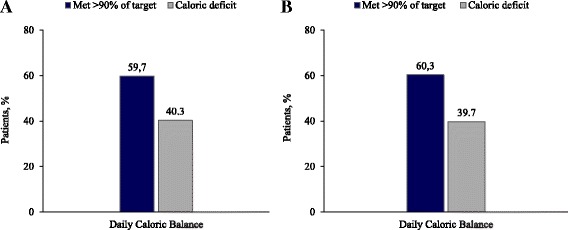
Daily caloric balance based on **a** clinician-derived daily target and **b** standardised daily target*. *Defined as the difference between the daily caloric target and the sum of calories from enteral and/or parenteral nutrition and other sources of caloric intake

The mean daily caloric balance on day –1 is presented in Table 3. Categorical analysis of daily caloric balance on day –1 showed that caloric intake met >90% of the daily target in 628 (59.7%) patients and resulted in a caloric deficit in 424 (40.3%) patients (Fig. 2). Among those who did not reach the caloric target, the mean caloric deficit on day –1 was –688.8 ± 455.2 kcal (–10.8 ± 7.0 kcal/kg). Sensitivity analysis using a standardised daily caloric target of 20 kcal/kg actual body weight (25 kcal/kg for patients with ≥ 6 days in the ICU) yielded similar results; caloric intake met >90% of the standardised daily caloric target in 607 (60.3%) patients and resulted in a caloric deficit in 399 (39.7%) patients. The mean caloric deficit among patients who failed to meet the standardised daily caloric target on day –1 was –640.3 ± 373.2 kcal (–9.5 ± 5.1 kcal/kg).

**Table 3:** Caloric balance on day –1^a^

Per protocol population (*N* = 1053)		
Caloric target^b^	Mean	Median
kcal	1609.1 (447.4)	1611.0 (1320.5; 1900.0)
kcal/kg	26.0 (7.3)	25.0 (21.5; 30.0)
Caloric intake	Mean	Median
kcal	1463.2 (683.1)	1465.5 (1000.0; 1800.0)
kcal/kg	24.0 (11.8)	23.9 (16.0; 30.1)
Caloric balance	Mean	Median
kcal	–146.1 (653.0)	–19.0 (–460.0; 71.5)
kcal/kg	–2.0 (10.6)	–0.2 (–7.1; 1.2)
Patients with caloric deficit (*n* = 424 [40.3%])		
Caloric target^b^	Mean	Median
kcal	1703.7 (446.5)	1700.0 (1400.0; 2000.0)
kcal/kg	26.7 (7.0)	25.0 (22.2; 30.0)
Caloric intake	Mean	Median
kcal	1014.9 (463.7)	1000.0 (700.0; 1327.5)
kcal/kg	15.8 (7.2)	15.8 (10.7; 20.8)
Caloric balance	Mean	Median
kcal	–688.8 (455.2)	–577.0 (–928.5; –327.5)
kcal/kg	–10.8 (7.0)	–9.2 (–14.9; –5.3)

*Abbreviations*: *Q1* lower quartile, *Q3* upper quartile, *SD* standard deviation

^a^Data are presented as mean (±SD) and median (Q1; Q3)

^b^Clinician-derived daily caloric target

**Fig. 3:**
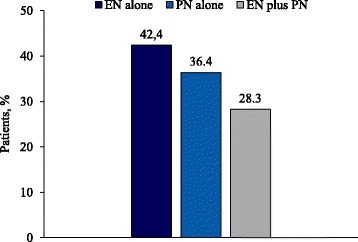
Proportion of patients with caloric deficit on day –1 according to prescribed nutritional therapy. *Abbreviations*: *EN* enteral nutrition, *PN* parenteral nutrition

Assessment of daily caloric balance according to the prescribed route of nutrition support showed that the proportion of patients with a caloric deficit was higher among those who received EN alone (42.4%) compared with either PN alone (36.4%) or a combination of EN and PN (28.3%) (Fig. 3).

Subgroup analysis according to nutritional status showed a lower incidence of caloric deficit on day –1 in patients who were classified as malnourished compared with those who were classified as well-nourished. Caloric deficits were observed in 29.5% and 43.1% of patients classified as malnourished and well-nourished, respectively, according to the ESPEN malnutrition score. Similarly, 36.7% of patients with suspected or moderate malnutrition according to the SGA had a caloric deficit on day –1, compared with 41.6% of patients with severe malnutrition and 47.3% of those who were well nourished. Assessment of daily caloric balance on day –1 according to the NUTRIC score, which measures the risk of death if not adequately fed, showed no meaningful differences between patients with a score indicating a high nutrition-related risk and those with a score indicating a low risk.

### Secondary outcomes

**Fig. 4:**
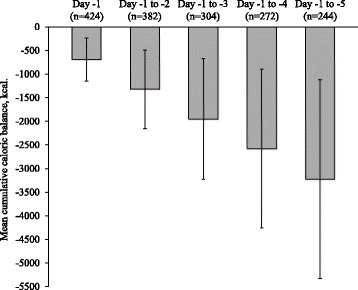
Mean (±SD) cumulative caloric balance in patients who did not meet the cumulative caloric target*. *Defined as the sum of the mean daily caloric balance for all days from day −1, calculated for each day from day −5 to day −1

Analysis of cumulative caloric balance from day –5 to day –1 showed a mean cumulative caloric deficit of –768.9 ± 2768.7 kcal (–11.1 ± 44.8 kcal/kg). Caloric intake during the 5-day observation period met >90% of the clinician-derived cumulative target in 60.1% of patients and failed to meet the cumulative caloric goal in 39.9%. Among the latter, caloric deficits accumulated rapidly, reaching a mean deficit of –3225 ± 2103 kcal (–50.6 ± 33.1 kcal/kg) for the period from day –5 to day –1 (Fig. 4).

**Fig. 5:**
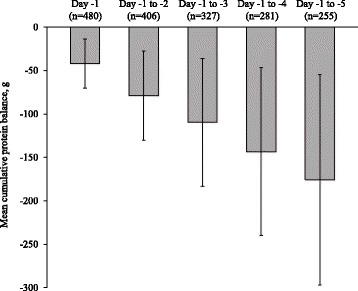
Mean (±SD) cumulative protein balance in patients who did not meet the cumulative protein target*. *Defined as the sum of the mean daily protein balance for all days from day −1, calculated for each day from day −5 to day −1

Protein intake on day –1 met >90% of the daily target in 52.4% of patients and failed to reach the daily target in 47.6%. The mean daily protein deficit on day –1 among patients for whom protein intake failed to meet the daily target was –42.2 ± 28.2 g (–0.7 ± 0.4 g/kg). Evaluation of the cumulative protein balance during the 5-day observation period showed that protein intake met >90% of the cumulative target in 53.9% of patients and failed to meet the cumulative target in 46.1%. The overall mean cumulative protein deficit for the period from day –5 to day –1 was –36.2 ± 216.5 g (–0.5 ± 3.4 g/kg). In patients who failed to meet the cumulative protein target, the mean cumulative protein deficit for the corresponding period was –175.7 ± 121.0 g (–2.7 ± 1.8 g/kg) (Fig. 5). Assessment of daily protein balance according to the prescribed route of nutrition support showed that the proportion of patients with a protein deficit was higher among those who received EN alone (50.3%) compared with either PN alone (37.4%) or a combination of EN and PN (36.2%).

Univariable (unadjusted) logistic regression analyses identified potential associations between attainment of daily caloric and protein targets on day –1 and various hospital and patient characteristics (Additional file 1: Appendix E). The results of the multivariable analyses are presented in Table 4. After adjustment for model covariates, the following variables were associated with a statistically significant increase in the probability of meeting >90% of the daily caloric target: female sex, BMI <18.5 kg/m^2^, suspected or moderate malnutrition according to the SGA, SOFA score ≥10, and combined administration of EN and PN. Variables associated with an increased probability of meeting >90% of the daily protein target included suspected or moderate malnutrition according to the SGA, administration of PN, and a duration of stay in the ICU before the Screening Day >10 days. The probability of meeting both the daily caloric target and the daily protein target was significantly increased among patients who received a combination of EN and PN as well as those with a >10-day duration of stay in the ICU before the Screening Day. Conversely, a BMI ≥30 kg/m^2^ was associated with a significantly lower probability of meeting either the daily caloric target or the daily protein target.

**Table 4:** Multivariable analysis of the association between reaching caloric and protein targets on day –1 and selected patient and hospital characteristics

Outcome	Variable	OR (95% CI)	*p* value^a^
Caloric target reached^b^	Sex		
	Male	–	–
	Female	1.39 (1.07, 1.82)	0.015
	Prescribed nutrition therapy		
	EN alone	–	–
	PN alone	1.30 (0.83, 2.04)	0.254
	EN plus PN	1.64 (1.04, 2.57)	0.032
	SGA score		
	A (well nourished)	–	–
	B (moderately malnourished)	1.40 (1.03, 1.91)	0.034
	C (severely malnourished)	0.92 (0.62, 1.36)	0.686
	SOFA score		
	<6	–	–
	6 to <10	1.25 (0.93, 1.67)	0.138
	≥10	1.85 (1.29, 2.65)	<0.001
	BMI, kg/m^2^		
	<18.5	2.07 (1.15, 3.74)	0.015
	18.5 to <20	1.58 (0.86, 2.92)	0.141
	20 to <22	1.02 (0.74, 1.69)	0.595
	22 to <30	–	–
	≥30	0.61 (0.43, 0.87)	0.006
Protein target reached^b^	Duration of ICU stay before day -1		
	0–5 days	–	–
	6–9 days	1.31 (0.88, 1.94)	0.181
	≥10 days	1.77 (1.30, 2.41)	<0.001
	Prescribed nutrition therapy		
	EN alone	–	–
	PN alone	1.79 (1.14, 2.81)	0.011
	EN plus PN	1.43 (0.92, 2.23)	0.110
	SGA score		
	A (well nourished)	–	–
	B (moderately malnourished)	1.54 (1.12, 2.12)	0.008
	C (severely malnourished)	0.88 (0.59, 1.32)	0.547
	BMI, kg/m^2^		
	<18.5	1.59 (0.91, 2.78)	0.102
	18.5 to <20	1.76 (0.97, 3.09)	0.064
	20 to <22	1.21 (0.80, 1.82)	0.373
	22 to <30	–	–
	≥30	0.45 (0.31, 0.64)	<0.001
Both caloric and protein target reached^b^	Duration of ICU stay before day -1		
	0–5 days	–	–
	6–9 days	1.38 (0.94, 2.03)	0.103
	≥10 days	1.69 (1.25, 2.29)	<0.001
	Prescribed nutrition therapy		
	EN alone	–	–
	PN alone	1.39 (0.91, 2.13)	0.132
	EN plus PN	1.56 (1.02, 2.39)	0.038
	BMI, kg/m^2^		
	<18.5	1.58 (0.94, 2.67)	0.084
	18.5 to <20	1.56 (0.89, 2.73)	0.120
	20 to <22	1.20 (0.81, 1.77)	0.373
	22 to <30	–	–
	≥30	0.49 (0.34, 0.70)	<0.001

*Abbreviations: BMI* body mass index, *CI* confidence interval, *EN* enteral nutrition, *OR* odds ratio, *PN* parenteral nutrition, *SGA* Subjective Global Assessment, *SOFA* Sequential Organ Failure Assessment

^a^Wald test

^b^ > 90% of daily target

Patient nutritional status and nutrition-related risk on day 0 are summarised in Table 5. Assessment of nutritional status using the SGA showed that 74.1% of patients were moderately to severely malnourished on the Screening Day. The ESPEN malnutrition score suggested the presence of malnutrition in 13.9% of patients. The NUTRIC score indicated a high need for nutritional therapy in 39.2%.

**Table 5:** Patient nutritional status and nutrition-related risk

Patients, n (%)		Total (*N* = 1053)
Nutritional status	Subjective Global Assessment (SGA)	
	Well nourished (A)	261 (25.9)
	Moderately malnourished (B)	512 (50.9)
	Severely malnourished (C)	233 (23.2)
	Missing	47
	ESPEN malnutrition score	
	Well nourished	816 (86.1)
	Malnourished^a^	132 (13.9)
	Missing	105
Nutrition-related risk	NUTRIC score	
	Low risk^b^	561 (60.8)
	High risk^c^	362 (39.2)
	Missing	130

*Abbreviations*: *BMI* body mass index, *ESPEN* European Society for Clinical Nutrition and Metabolism; *FFMI* fat-free mass index, *IL-6* interleukin-6, *NUTRIC* Nutrition Risk in the Critically Ill

^a^Defined as (a) BMI <18.5 kg/m^2^ or (b) combination of unintentional weight loss (>10% over an undefined time period or >5% during previous 3 months) and either low BMI (<20 kg/m^2^ [age <70 years] or <22 kg/m^2^ [age ≥70 years]) or low FFMI (<15 kg/m^2^ [women] or <17 kg/m^2^ [men])

^b^Score of 0–5 for patients with available IL-6 concentration or 0–4 for patients with no available IL-6 concentration

^c^Score of 6–10 for patients with available IL-6 concentration or 5–9 for patients with no available IL-6 concentration

More than 90% of patients received EN during the observation period; of these, 88.2% received EN alone and 11.8% received EN in combination with PN. Intestinal failure precluded initiation of EN in 17.7% of all patients and intolerance to EN was observed in 18.7%. Diarrhoea, high gastric residual volume, and abdominal distention were the most commonly reported causes of intolerance to EN. Nutrition therapy was interrupted during the 5-day observation period in 25.7% of patients; the most common reasons for interruption were intolerance (43.1%), diagnostic procedures (32.2%), and surgery (27.8%).

## Discussion

The present study represents the first large multinational study evaluating nutrition practices and in adult critically ill ICU patients in Latin America. Analysis of data from 1053 patients in 116 hospitals in eight Latin American countries yielded several observations with important implications for the development of improved nutrition practices in patients with critical illness.

First, malnutrition is highly prevalent in critically ill adult patients in Latin America. More than 70% of all patients had moderate or severe malnutrition according to the SGA and nearly 40% had an increased risk of poor clinical outcomes according to the NUTRIC score. The ESPEN malnutrition score suggested the presence of malnutrition in only 13.9%. To our knowledge, the current study is the first to apply the ESPEN diagnostic criteria to patients with critical illness. The observed difference in the proportion of patients classified as malnourished according to the SGA and the ESPEN criteria is likely attributable to the fact that the ESPEN definition does not account for the influence of disease severity or the increased metabolic demands in critically ill patients. The proportion of patients who were malnourished according to the SGA is consistent with a Brazilian study in which the reported prevalence of malnutrition among ICU patients with a duration of hospitalisation >48 hours was 70.3% [19]. In that study, malnourished patients had a significantly higher rate of readmission to the ICU (OR 2.27; 95% CI 1.08–4.80) and a markedly increased risk of mortality compared with well-nourished patients (OR 8.12; CI 2.94–22.42). Coupled with these findings, the observed prevalence of malnutrition in the present study underscores the need for adequate nutritional screening and assessment, prompt intervention, and rigorous monitoring of nutritional status in this high-risk population.

Second, caloric intake failed to meet the established daily target in 40% of patients on day –1, and a similar proportion of patients failed to achieve the caloric target during the 5-day observation period. Moreover, caloric deficits accumulated rapidly in these patients, resulting in a mean caloric deficit of –3225 kcal between day –5 and day –1. This latter finding is particularly alarming, as caloric deficits in critically ill patients are associated with poor clinical outcomes, including infectious complications, prolonged duration of mechanical ventilation, and increased mortality [14, 27, 28]. Importantly, emerging evidence suggests that low but adequate caloric intake during the first few days following ICU admission may confer benefits to critically ill patients, particularly when coupled with increased protein intake [11, 12]. Of note, sensitivity analysis of caloric balance using a caloric target of 20 kcal/kg/d for the first 6 days in the ICU and 25 kcal/kg/d thereafter showed no meaningful difference in the observed proportion of patients with a caloric deficit.

Third, while more than 90% of patients received EN, PN use was low. Fewer than one in ten patients received PN alone and slightly more than 10% received a combination of EN and PN. This finding is consistent with the recent Nutrition Day ICU survey in which 10% and 12% of patients received PN and combined EN and PN, respectively [29]. In contrast to the limited use of PN observed during the present study, the clinical characteristics of the population suggested a need for broader use. More than 75% of patients required mechanical ventilation, 74% had suspected/moderate or severe malnutrition according to the SGA, and more than one-third of all patients had either a contraindication or intolerance to EN. Notably, patients who received EN alone had a larger mean daily caloric deficit on day –1 compared with those who received either PN or a combination of EN and PN, and a higher proportion of patients who received EN alone failed to meet at least 90% of the daily caloric target compared with those who received PN or combined EN and PN. A previous randomised controlled trial of 305 critically ill patients with persistent energy deficits following 3 days of EN showed that supplemental administration of PN improved the cumulative energy balance compared with continued administration of EN alone [30]. The effects of supplemental PN and caloric intake on clinical outcome parameters are discussed controversially [30–36]. However, current recommendations indicate that persistent energy and protein deficits for 2 days (ESPEN guidelines) or 7–10 days (A.S.P.E.N. guidelines) in patients receiving EN alone should prompt the clinician to consider the use of supplemental PN to improve energy and protein delivery and potentially reduce the risk of adverse clinical outcomes [11, 12]. The results of the present study show that supplemental PN reduces energy and protein deficits but suggest that the supplemental use of PN is not optimally employed as a therapeutic strategy in patients who fail to receive adequate nutrition intake from EN alone.

Finally, logistic regression analysis identified a statistically significant association between achieving >90% of the targeted energy and protein delivery on day –1 and the type of prescribed nutritional therapy. Combined administration of EN and PN was associated with a 64% increase in the likelihood of meeting >90% of the daily caloric target on day –1 and a 56% increase in the probability of meeting >90% of both the daily caloric target and the daily protein target compared with EN alone. Given the relatively low use of PN in the present study and the observed improvements in cumulative energy balance among ICU patients receiving supplemental PN in a previous randomised trial [30], the association between supplemental PN and caloric and protein target attainment in the present study suggests an opportunity to improve energy and protein delivery in critically ill patients in Latin America through the incorporation of supplemental PN into the routine nutritional management plan. Common practice in Latin America is to use EN before adding or switching to PN whenever EN is not contraindicated. In view of this general approach of dynamically adapting EN and PN (including SPN) to meet individual nutritional requirements, the current results clearly suggest an opportunity for further improvements in optimising nutrition delivery to reach defined nutritional targets.

The strengths of our study include the large sample size and the high rate of data ascertainment for the parameters of interest. Additionally, in contrast to studies in which patients share a common starting point at the time of ICU admission, the design of the present study allowed for a representative mix of patients with different durations of ICU stay before the time of the assessment. Moreover, the study population included a high proportion of patients with a long duration of ICU stay, thereby facilitating assessment of the relationship between longer duration of stay and prescribed nutrition therapy.

The findings of our study are subject to certain limitations, including those inherent to cross-sectional study design and retrospective analysis. Additionally, due to lower than expected recruitment on the Screening Day, the sample size was smaller than the originally planned sample size of 2000 patients. Based on an estimated 50% prevalence of malnutrition, the original calculation showed that a sample size of 2000 patients would provide a confidence interval with a precision of ±2.2%. Recalculation based on the observed 74% prevalence of malnutrition and the actual sample size of 1053 patients yielded a confidence interval with a precision of ±2.7%. The marginal difference in the precision of the confidence intervals between sample sizes of 1053 and 2000 patients (0.5%) suggests that the smaller sample size had a negligible effect on the precision of the estimates. A substantial majority of hospitals in the study were academic institutions (83.7%) and 39.7% of the ICUs had a specialised nutritional team; accordingly, the extent to which the findings are generalisable to local/community hospitals is uncertain. Moreover, patients were required to be receiving nutrition therapy on both the Screening Day and the previous day; therefore, the degree to which nutrition therapy is optimally employed across the full population of critically ill patients in the ICU setting cannot be reliably ascertained. Finally, both the prevalence of underfeeding and the magnitude of caloric deficits observed in the present study were lower than those reported in a recent prospective study in nutritionally at-risk critically ill patients [37]. This might be explained in part by the cross-sectional design of the present study, which resulted in a longer pre-screening duration of ICU stay. The median duration of stay in the ICU on the Screening Day was 10 days, reflecting a mixed population of acute and chronically ill patients. The previous study [37] evaluated patients beginning 96 hours after admission to the ICU and therefore included a larger proportion of patients in the acute phase of illness. Other potential explanations for the observed differences include a lower proportion of patients with gastrointestinal intolerance, a lower proportion of patients requiring invasive respiratory support, a higher proportion of hospitals with nutrition therapy teams, and a higher proportion of patients who received PN compared with the previous study. However, despite these differences, the results of the two studies are directionally consistent and provide compelling corroborative evidence of the need for improved nutrition practices to optimise energy provision in critically ill patients.

## Conclusions

Comprehensive assessment of the nutritional status of critically ill adults receiving EN and/or PN in Latin American hospitals identified caloric deficits in more than 40% of patients on EN, with lower deficits observed in patients receiving a combination of EN and PN. Coupled with the low rates of PN use observed in the study, these data suggest an opportunity for more effective utilisation of supplemental PN in critically ill adults who fail to receive adequate nutrition intake from EN alone.

## Additional files

Additional file 1: **Appendix A** Subjective Global Assessment (SGA) Questionnaire. **Appendix B** Nutrition Risk in the Critically Ill (NUTRIC) Score. **Appendix C** ESPEN Diagnostic Criteria for Adult Malnutrition. **Appendix D** Hospital characteristics. **Appendix E** Univariable analyses—association between daily caloric and protein balance and selected hospital and patient characteristics. **Appendix F** Screening Day Latin America investigators and study sites. (PDF 371 kb)

Additional file 2: Ethics committee approvals. (PDF 225 kb)

## Abbreviations

APACHE II: Acute Physiology and Chronic Health Evaluation II
A.S.P.E.N.: American Society for Parenteral and Enteral Nutrition
BMI: Body mass index
EN: Enteral nutrition
ESPEN: European Society for Clinical Nutrition and Metabolism
ICU: Intensive care unit
NUTRIC: Nutrition Risk in the Critically Ill
PN: Parenteral nutrition
SD: Standard deviation
SGA: Subjective Global Assessment
SOFA: Sequential Organ Failure Assessment
