# Supplementary material for: Current clinical nutrition practices in critically ill patients in Latin America: a multinational observational study
Source: Crit Care. 2017 Aug 25;21:227. doi: 10.1186/s13054-017-1805-z (PMC6389103; doi:10.1186/s13054-017-1805-z)
Supplement: Supplementary file 1 — Appendix A Subjective Global Assessment (SGA) Questionnaire. Appendix B Nutrition Risk in the Critically Ill (NUTRIC) Score. Appendix C ESPEN Diagnostic Criteria for Adult Malnutrition. Appendix D Hospital characteristics. Appendix E Univariable analyses—association between daily caloric and protein balance and selected hospital and patient characteristics. Appendix F Screening Day Latin America investigators and study sites. (PDF 371 kb) [file 13054_2017_1805_MOESM1_ESM.pdf]

Current Clinical Nutrition Practices in Critically Ill Patients in Latin America: A Multinational Observational Study

Supplemental Material

Appendix A Subjective Global Assessment (SGA) Questionnaire

Appendix B Nutrition Risk in the Critically Ill (NUTRIC) Score

Appendix C ESPEN Diagnostic Criteria for Adult Malnutrition

Appendix D Hospital Characteristics

Appendix E Univariable Analyses—Association between Daily Caloric and Protein Balance and Selected Hospital and Patient Characteristics

Appendix F Screening Day Latin America Investigators and Study Sites

Appendix A

Subjective Global Assessment (SGA) Questionnaire[1]

A. History

1. Weight change

   Overall loss in past 6 months: ___kg ___% loss
   Change in past 2 weeks: increase, no change, decrease

2. Dietary intake change relative to normal

   No change
   Change: duration ___weeks
   Type: sub-optimal solid diet, full liquid diet, hypocaloric liquid, starvation

3. Gastrointestinal symptoms (persisted for >2weeks)

   None
   Nausea
   Vomiting
   Diarrhea
   Anorexia

4. Functional Capacity

   No dysfunction
   Dysfunction: duration ___weeks
   Type: working sub-optimally, ambulatory, bedridden

5. Disease and its relationship to nutritional requirements

   Primary diagnosis: ________________________
   Metabolic demand/stress: no, low, moderate, high

B. Physical (for each specify: 0=normal, 1+=mild, 2+=moderate, 3+=severe)

___ Loss of subcutaneous fat (triceps, chest)
___ Muscle wasting (quadriceps, deltoids)
___ Ankle edema
___ Sacral edema
___ Ascites

C. Subjective Global Assessment Rating

Well-nourished	A
Suspected or moderately malnourished	B
Severely malnourished	C

1. Detsky AS, McLaughlin JR, Baker JP et al. What is subjective global assessment of nutritional status? *J Parenter Enteral Nutr* 1987;11:8–13.

Appendix B

Nutrition Risk in the Critically Ill (NUTRIC) Score[1]

Clinical Variables

Variable	Range	Points
Age, years	<50<br>50 to <75<br>≥75	0<br>1<br>2
APACHE II score	<15<br>15 to <20<br>20 to <28<br>≥28	0<br>1<br>2<br>3
SOFA score	<6<br>6 to <10<br>≥10	0<br>1<br>2
Number of co-morbidities	0 to 1<br>≥2	0<br>1
Days from hospital to ICU admission	0 to <1<br>≥1	0<br>1
IL-6 concentration, [μ/mL]	0 to <400<br>≥400	0<br>1

Abbreviations: APACHE=acute physiology and chronic health evaluation; ICU= intensive care unit; IL6=interleukin-6; SOFA=sequential organ failure assessment

Scoring system if IL-6 is available

Sum of points	Category	Explanation
6-10	High Score	– Associated with worse clinical outcomes (mortality, ventilation)<br>– These patients are most likely to benefit from aggressive nutrition therapy
0-5	Low Score	– These patients have a low malnutrition risk

Scoring system if IL-6 is not available

Sum of points	Category	Explanation
5-9	High Score	– Associated with worse clinical outcomes (mortality, ventilation)<br>– These patients are most likely to benefit from aggressive nutrition therapy
0-4	Low Score	– These patients have a low malnutrition risk

1. Heyland DK, Dhaliwal R, Jiang X, Day AG. Identifying critically ill patients who benefit the most from nutrition therapy: The development and initial validation of a novel risk assessment tool. *Critical Care* 2011;15:R268.

Appendix C

ESPEN Diagnostic Criteria for Adult Malnutrition[1]

Malnutrition defined as:

(a) BMI <18.5 kg/m^2

or

(b) combination of:

unintentional weight loss (>10% over undefined period or >5% during previous 3 months) and either of the following:

- low BMI (<20 kg/m^2 [age <70 years] or <22 kg/m2 [age ≥70 years])
- low FFMI (<15 kg/m^2 [women] or <17 kg/m^2 [men])

Abbreviations: BMI=body mass index; FFMI=fat free mass index

1. Cederholm T, Bosaeus I Barazzoni R, et al. Diagnostic criteria for malnutrition—An ESPEN Consensus Statement. *Clin Nutr* 2015;34:335–340.

Appendix D

Hospital Characteristics

**Characteristic, n (%)**	**Total (N=116)**
Type, n (%)	
Academic/university	94 (81.0)
Local/regional	18 (15.5)
Unspecified	4 (3.4)
Care level, n (%)	
Tertiary	93 (80.2)
Secondary	21 (18.1)
Unspecified	2 (1.7)
Financing/ownership, n (%)	
Public	58 (50.0)
Private	37 (31.9)
Public-private	13 (11.2)
Other	3 (2.6)
Unspecified	5 (4.3)
Hospital size, n (%)	
≤300 beds	56 (48.3)
300 to ≤600 beds	38 (32.8)
>500 beds	19 (16.4)
Unspecified	3 (2.6)
Total beds, mean (SD)	388.1 (299.3)
ICU size, n (%)	
≤25 beds	71 (61.2)
25 to ≤50 beds	31 (26.7)
>50 beds	12 (10.3)
Unspecified	2 (1.7)
ICU beds, mean (SD)	27.8 (28.7)
Type of nutrition provided, n (%)*	
Oral	100 (86.2)
Enteral	101 (87.1)
Parenteral	101 (87.1)
Combination	112 (96.6)

Type of enteral access, n (%)*	
Nasogastric	114 (98.3)
Postpyloric	102 (87.9)
Nasoduodenal	66 (56.9)
Nasojejunal	83 (71.6)
Percutaneous endoscopic gastrostomy (PEG)	103 (88.8)
Percutaneous endoscopic jejunostomy (PEJ/FCJ)	54 (46.6)
Type of venous access, n (%)*	
Central	114 (98.3)
Jugular	91 (78.4)
Subclavian	107 (92.2)
Femoral	33 (28.4)
Peripheral	70 (60.3)
Peripherally inserted central catheter	46 (39.7)
Nutritional support team, n (%)	
Hospital	44 (37.1)
Academic/university	38 (86.4)
Local/regional	5 (11.4)
Unspecified	1 (2.2)
ICU	46 (39.7)
Academic/university	36 (78.3)
Local/regional	7 (15.2)
Unspecified	3 (6.5)
Responsibility for enteral nutrition, n (%)*	
Hospital	
Physician	80 (70.8)
Dietitian	58 (51.3)
Nutritional team	63 (55.8)
Other	5 (4.4)
ICU	
Physician	85 (74.6)
Dietitian	47 (41.2)
Nutritional team	48 (42.1)
Other	7 (6.1)

Responsibility for parenteral nutrition, n (%)*	
Hospital	
Physician	78 (68.4)
Dietitian	28 (24.6)
Nutrition team	49 (43.0)
Other	7 (6.1)
ICU	
Physician	83 (73.5)
Dietitian	26 (23.0)
Nutrition team	45 (39.8)
Other	8 (7.1)
Nutrition Guidelines, n (%)*	
ESPEN	75 (64.7)
ASPEN	75 (64.7)
FELANPE	45 (38.8)
Local guidelines	44 (37.9)
Hospital operational policy/standards	48 (41.4)
Other	20 (17.2)

*Sum of percentages may exceed 100% due to the ability to select more than one option.

Abbreviations: ASPEN=American Society for Parenteral and Enteral Nutrition; ESPEN= European Society for Clinical Nutrition and Metabolism; FELANPE=Latin American Federation for Nutritional Therapy Clinical Nutrition and Metabolism; ICU=intensive care unit; SD=standard deviation

Appendix E

Univariable Analyses—Association between Daily Caloric and Protein Target Attainment and Selected Hospital and Patient Characteristics*

	Caloric Target		Protein Target		Caloric + Protein Target	
**Variable**	**OR (95% CI)**	**p-value†**	**OR (95% CI)**	**p-value†**	**OR (95% CI)**	**p-value†**
**Gender**		**0.068**		**0.655**		**0.194**
Male	–	–	–	–	–	–
Female	1.26 (0.98–1.62)	0.068	1.06 (0.82–1.36)	0.655	1.18 (0.92–1.52)	0.194
**Age, years**		**0.676**		**0.747**		**0.578**
18–54	–	–	–	–	–	–
55–64	0.93 (0.65–1.31)	0.667	1.11 (0.79–1.58)	0.547	1.15 (0.81–1.63)	0.433
65–74	0.84 (0.60–1.18)	0.327	0.89 (0.63–1.24)	0.484	0.84 (0.60–1.19)	0.324
75–84	0.79 (0.55–1.14)	0.209	1.02 (0.71–1.46)	0.925	0.91 (0.63–1.30)	0.599
≥85	0.78 (0.46–1.31)	0.344	0.81 (0.47–1.39)	0.447	0.86 (0.50–1.49)	0.595
**Leading diagnosis at admission**		**0.475**		**0.368**		**0.312**
Locomotive system	–	–	–	–	–	–
Cardiac	0.86 (0.40–1.82)	0.688	1.09 (0.51–2.31)	0.826	0.85 (0.40–1.79)	0.661
Digestive	1.03 (0.54–1.95)	0.926	1.22 (0.65–2.31)	0.538	1.12 (0.60–2.11)	0.723
Endocrine	0.49 (0.18–1.36)	0.173	1.03 (0.36–2.95)	0.950	0.89 (0.31–2.53)	0.826
Genitourinary	1.02 (0.34–3.03)	0.975	0.95 (0.31–2.88)	0.931	1.22 (0.40–3.70)	0.722
Renal	1.10 (0.45–2.65)	0.838	1.00 (0.42–2.41)	0.991	1.03 (0.43–2.45)	0.953
Hematological	0.89 (0.22–3.56)	0.870	1.27 (0.32–5.08)	0.736	1.63 (0.41–6.52)	0.489
Oncology	0.79 (0.36–1.72)	0.555	0.70 (0.32–1.53)	0.373	0.71 (0.32–1.57)	0.402
Neurological	0.98 (0.53–1.82)	0.960	0.96 (0.52–1.76)	0.887	0.99 (0.54–1.82)	0.969
Respiratory	0.68 (0.36–1.28)	0.232	0.69 (0.37–1.31)	0.256	0.67 (0.36–1.28)	0.227
Vascular	0.50 (0.19–1.34)	0.170	0.51 (0.19–1.38)	0.185	0.36 (0.12–1.07)	0.066
Other	0.65 (0.28–1.50)	0.314	1.14 (0.49–2.67)	0.754	0.98 (0.42–2.28)	0.969
**Duration of ICU stay before Day 0**		**0.003**		**0.002**		**<0.001**
0–5 days	–	–	–	–	–	–
6–9 days	1.28 (0.89–1.85)	0.180	1.35 (0.93–1.96)	0.112	1.34 (0.92–1.95)	0.132
≥10 days	1.65 (1.24–2.19)	<0.001	1.68 (1.26–2.25)	<0.001	1.74 (1.30–2.33)	<0.001
**Prescribed nutrition therapy**		**0.013**		**0.003**		**0.009**
EN alone	–	–	–	–	–	–
PN alone	1.29 (0.84– 1.98)	0.252	1.70 (1.10–2.61)	0.016	1.34 (0.88–2.04)	0.166
EN plus PN	1.86 (1.21– 2.87)	0.005	1.79 (1.17–2.72)	0.007	1.83 (1.21–2.76)	0.004

**SGA score**		**0.017**		**<0.001**		**0.025**
A (well nourished)	0.79 (0.56–1.13)	0.206	0.76 (0.53–1.09)	0.140	0.75 (0.52–1.08)	0.123
B (moderately malnourished)	1.23 (0.90–1.69)	0.201	1.40 (1.02–1.91)	0.036	1.15 (0.84–1.58)	0.371
C (severely malnourished)	–	–	–	–	–	–
**NUTRIC score**		**0.718**		**0.402**		**0.390**
High risk	1.04 (0.79–1.36)	0.718	0.89 (0.68– 1.17)	0.402	0.89 (0.68–1.16)	0.390
Low risk	–	–	–	–	–	–
**APACHE II score**		**0.833**		**0.878**		**0.882**
<15	–	–	–	–	–	–
15 to <20	0.92 (0.67–1.26)	0.609	1.05 (0.76–1.44)	0.784	1.08 (0.78–1.48)	0.654
20 to <28	1.00 (0.73–1.36)	0.977	1.08 (0.79–1.48)	0.615	1.01 (0.74–1.39)	0.926
≥28	1.14 (0.74–1.74)	0.557	0.91 (0.60–1.39)	0.666	0.90 (0.59–1.37)	0.612
**SOFA score**		**0.022**		**0.741**		**0.316**
<6	–	–	–	–	–	–
6 to <10	1.16 (0.88–1.53)	0.283	1.03 (0.78–1.36)	0.840	1.16 (0.88–1.53)	0.300
≥10	1.63 (1.15–2.30)	0.006	1.14 (0.82–1.60)	0.441	1.27 (0.91–1.78)	0.158
**BMI, kg/m^2**		**0.002**		**<0.001**		**<0.001**
<18.5	1.89 (1.08–3.32)	0.026	1.51 (0.89–2.56)	0.123	1.71 (1.02–2.86)	0.042
18.5 to <20	1.57 (0.87–2.84)	0.133	1.52 (0.86–2.69)	0.151	1.54 (0.88–2.67)	0.129
20 to <22	1.08 (0.73–1.60)	0.687	1.13 (0.76–1.67)	0.539	1.19 (0.81–1.75)	0.379
22 to <30	–	–	–	–	–	–
≥30	0.64 (0.46–0.89)	0.008	0.46 (0.33–0.66)	<0.001	0.49 (0.34–0.70)	<0.001
**Hospital type**		**0.852**		**0.458**		**0.382**
Local/regional	–	–	–	–	–	–
Academic/university	1.04 (0.70–1.53)	0.852	0.86 (0.58–1.28)	0.458	0.84 (0.57–1.24)	0.382
**Co-morbidities, n**		**0.119**		**0.166**		**0.328**
0	–	–	–	–	–	–
1–5	1.14 (0.79–1.64]	0.489	1.06 (0.74–1.54)	0.740	1.02 (0.71–1.49)	0.897
5	0.57 (0.27–1.20)	0.139	0.52 (0.23–1.16)	0.112	0.57 (0.25–1.31)	0.185

*Variables with a p-value ≤0.20 selected for inclusion in the multivariable analysis.

†Wald test

Abbreviations: APACHE=acute physiology and chronic health evaluation; BMI=body mass index, CI=confidence interval, EN=enteral nutrition, ICU=intensive care unit; NUTRIC=nutrition risk in the critically ill; OR=odds ratio, PN=parenteral nutrition, SGA=subjective global assessment, SOFA=sequential organ failure assessment

Appendix F

Screening Day Latin America Investigators and Study Sites

Country	Investigator	Institution	City
Argentina	Elvis Eglyn Salcedo Noriega	Sanatorio Colegiales	Ciudad Autónoma de Buenos Aires
	Fernando Lipovestky	UAI Salud Hospital Universitario	Ciudad Autónoma de Buenos Aires
	Julia Rodríguez Bugueiro	Hospital de Alta Complejidad El Cruce Dr. Néstor Carlos Kirchner	Florencio Varela, Pcia. de Buenos Aires
	Cecilia Loudet, Leandro Tumino	Hospital Interzonal General de Agudos General de San Martín	La Plata, Pcia. de Buenos Aires
	Federico Viano, Victoria González	Sanatorio Allende	Córdoba
	Martín Buncuga	Hospital Eva Perón	Granadero Baigorria, Pcia. de Santa Fé
	Paulina Astegiano	Hospital Dr. José María Cullen	Santa Fé
	Raúl Valdez	Hospital Centro de Salud Zenón J. Santillán	San Miguel de Tucumán
	Sebastián Chapela	Hospital Británico	Ciudad Autónoma de Buenos Aires
	Mariana Di Sibio	Hospital Ramón Carrillo	Ciudadela, Pcia. de Buenos Aires
	Daniela Coronado	Hospital Raúl Ferreyra	Córdoba
	Andrés Martinuzzi	CMIC	Neuquén
	María Alejandra Peretti, Virginia Pamparana	Hospital Dr. Arturo Oñativia	Salta
	Jorge Audisio	Hospital Interzonal General Dr. José Penna	Bahía Blanca, Pcia. de Buenos Aires
	Martín Ayuso	Fundación Médica de Bahía Blanca	Bahía Blanca, Pcia. de Buenos Aires
	Giselle Sutton	Sanatorio San José	Ciudad Autónoma de Buenos Aires
	Fernando Baccaro	Hospital General de Agudos Dr. Juan A. Fernández	Ciudad Autónoma de Buenos Aires
	Maria Laura Cabana	Hospital Pablo Soria	San Salvador de Jujuy
	Maria Laura Cabana	Instituto Médico del Norte	San Salvador de Jujuy
Brazil	Simone Chaves de Miranda Silvestre	Hospital Felício Rocho	Belo Horizonte
	Maria Isabel Toulson Davisson Correia	Hospital das Clinical Minas Gerais	Belo Horizonte
	Bianca Zanchetta Buani Miguel	Hospital de Caridade São Vicente de Paulo	Jundiai
	Paulo Cesar Ribeiro	Sociedade Beneficente de Senhoras Hospital Sírio-Libanês	Sao Paulo
	Maria Angela de Souza	Instituto de Assistência Médica ao Servidor Público Estadual	Sao Paulo
	Maria de Lourdes Teixeira da Silva	Hospital Beneficência Portuguesa de SP	Sao Paulo
	Dan Linetzky Waitzberg	Hospital das Clinical da Faculdade de Medicina da USP	Sao Paulo
	Gilmária Millere Tavares	Hospital Metropolitano	Serra
	Luiz Stanislau Nunes Chini	Hospital Universitario Antonio Pedro	Niteroi
	Cristiane da Silva Belo	Rede D'Or Sao Luiz S.A.	Rio de Janeiro
	Andre Ney Menezes Freire	Santa Casa de Misericordia da Bahia	Salvador
	Gilmária Millere Tavares	Assoc. Sta Catarina- Hospital Central - Benicio Tavares	Serra
	Gilmária Millere Tavares	Unimed Vitoria Cooperativa de Trabalho Medico	Serra
	Marcelo Cassio de Souza	Hospital Santa Marcelina de Itaquera	Sao Paulo
Chile	Bárbara Valenzuela	Hospital Dipreca	Santiago

	Pilar Lora, María Eugenia Jeria	Hospital Santiago Oriente Dr. Luis Tisné Brousse	Santiago
	Joselyn Rubio, Karina Andrea Gonzalez Cuevas	Hospital Dr. Cesar Garavagno Burotto	Talca
	Álvaro Morales	Hospital San Borja – Arriarán	Santiago
	Jean Baptiste Camousseigt	Hospital del Salvador	Providencia
	Sebastián Ugarte, Pamela Hermosilla	Clínica Indisa	Providencia
	Julieta Klaassen	Hospital Clínico Universidad Católica de Chile	Santiago
	Gonzalo García	Clínica Dávila	Santiago
	Marylucy Galvez	Hospital San José	Santiago
	Daniel Morales	Hospital Metropolitano de la Florida Dra Eloísa Diaz Insunza	Santiago
	Jaime Vidal	Hospital Naval Almirante Nef	Viña del Mar
	Ana María Molina	Hospital Militar de Santiago	La Reina
	Elena Canales	Clínica Bicentenario	Santiago
	Karin Papapietro, Emma Díaz	Hospital Clínico Universidad de Chile	Santiago
	Claudia Vicuña	Complejo Asistencial Barros Luco	Santiago
	Daniela Ponce	Guillermo Grant Benavente	Concepción
	Susanne Ferrand	Hospital Dr. Gustavo Fricke	Viña del Mar
	Carolina Concha	Hospital Las Higueras	Concepción
	Mauricio Cortés	Hospital Dr. Hernán Henríquez Aravena	Temuco
	Sofía Araya, Maritza Aceituno	Hospital Regional de Antofagasta	Antofagasta
	Darwin Acuña	Hospital Clínico Mutual De Seguridad	Santiago
Colombia	Jorge Lemus	Clínica De La Costa	Barranquilla
	Francisco Ayola, Rafael Tejera	Hospital Universitario Cari E.S.E.	Barranquilla
	Mauricio Chona, Lina López	Clínica Colombia	Bogotá
	Nestor Muñoz	Hospital El Tunal E.S.E.	Bogotá
	Claudia Medina	Sociedad De Cirugía De Bogotá Hospital De San José	Bogotá
	Claudia Vacca	Hospital Universitario Clínica San Rafael	Bogotá
	Olga Pinzón, Luz Jeannette Romero	Hospital Universitario Mayor Mederi	Bogotá
	Juan Gabriel Bayona, Ana María Ortiz	Fundación Valle De Lili	Cali
	Raúl Escobar	Clínica San Jose De Cúcuta	Cúcuta
	Holmes Algarín	Ips Unipamplona	Cúcuta
	Jairo Figueroa	Hospital Universitario Erasmo Meoz	Cúcuta
	Mónica Yepes, Oscar Villada	Hospital Universitario San Vicente Fundación	Medellín
	Beatriz Restrepo, María Cecilia Sepúlveda	Hospital General De Medellín Luz Castro De Gutiérrez	Medellín
	Jessica Hernández, Yenny Díaz	Clínica Foscal Internacional - Fosunab	Floridablanca
	Janeth Barbosa	Clínica Las Américas	Medellín
	Gloria Chaves	Hospital Universitario San José de Popayán	Popayán
	Yenny Díaz	Fundación Oftalmológica De Santander – Foscal	Floridablanca
	Agamenón Quintero	Oncomedica S.A IMAT	Montería
	Orlando Villarreal, Yuly Pauline Mafioly	Evaluamos Ips	Montería
	María Eugenia Varela	S.E.S Hospital De Caldas	Manizales

	Luisa Fernanda Torres	Clínica Rey David	Cali
	Olga Mejía	Clínica Medellín	Medellín
Ecuador	Mario Arboleda	Hospital Eugenio Espejo	Quito
	Bayron Salgado	Hospital de los Valles	Quito
	Paula Salazar	Hospital IESS San Francisco	Quito
	Mario Vargas	Hospital Metropolitano	Quito
	Fernando Erazo	Hospital de la Policía	Quito
	Celia Luna	Hospital Luis Vernaza	Guayaquil
	Loni Bernabé	Hospital IESS Teodoro Maldonado Carbo	Guayaquil
	John Cuenca	Hospital Clínica Kennedy Alborada	Guayaquil
	Miguel Chung Sang	Hospital Universitario	Guayaquil
	Fernando Ortega	Hospital del Río	Cuenca
	Marcelo Ochoa	Hospital del IESS José Carrasco Arteaga	Cuenca
	Bolívar Guevara	Hospital Carlos Andrade Marín	Quito
	Miguel Llano	Hospital de las Fuerzas Armadas	Quito
	Miguel Llano	Hospital Inglés	Quito
Mexico	Vanessa Fuchs Tarlovsky	Hospital General de México	Ciudad de México
	Gabriel Mejía Consuelos	Hospital General Balbuena	Ciudad de México
	Liliana Navarro Gallo	Hospital Civil de Guadalajara	Guadalajara
	Dulce María Déctor Lira	Centro Médico Dalinde	Ciudad de México
	Carolina Oaxaca Ortega	Hospital General Pachuca	Pachuca
	Gabriela Tobalina Legaspi	CAE Centro de Alta Especialidad Dr. Rafael Lucio	Xalapa
	César Emilio Flores Santos	Hospital Regional de Veracruz	Veracruz
	Viridiana García Farfán	Centro Médico Chiapas Nos Une “Dr. Jesús Gilberto Gómez Maza”	Tuxtla Gutiérrez
	Maribel Peña Corona	ISEM CENTRO MÉDICO	Toluca de Lerdo
	Karla Fernández Villalobos	Central Universitario	Chihuahua
	Carlos Tadeo Perzabal Avilez	Hospital General de Cd. Juárez	Ciudad Juárez
	Isabel Guadalupe Calvo Higuera	Hospital General de Tijuana	Tijuana
	Patricia Rosalía Ancer Rodríguez	Hospital Universitario Dr. José Eleuterio González	Monterrey
	Laura Matienzo Valle	Hospital General de Soledad de Graciano Sánchez	San Luis Potosi
	Juana María Cerda Arteaga	Hospital Metropolitano “Dr. Bernardo Sepúlveda”	San Nicolás de los Garza
Panama	Alfredo Matos Adames	Complejo Hospitalario de la Caja del Seguro Social de Panamá	Ciudad de Panamá
Peru	José Portugal Sánchez	Hospital Nacional Edgardo Rebagliatti Martins	Lima
	Sergio Echenique Martinez	Hospital Nacional Guillermo Almenara Irigoyen	Lima
	Juan Carlos Plácido Olivos	Hospital Santa Rosa	Lima
	Teresa Samamé	Hospital de la Policía	Lima
	Manuel Laca Barrera	Hospital Naval	Callao
	Nelsa Pacheco	Hospital Nacional Arzobispo Loayza	Lima
	Humberto Lira	Complejo Hospitalario Alberto Leopoldo Barton Thompson	Lima
	Keith del Águila	Clínica Centenario Peruano Japonesa	Lima

	Mario Castañeda	Hospital Alberto Sabogal Sologuren	Callao
	Humberto Lira	Hospital Nacional Dos de Mayo	Lima
	Noemí Sosa	Hospital Víctor Lazarte Echegaray	Trujillo
	Guillermo Contreras	Complejo Hospitalario Guillermo Kaelin de la Fuente	Villa María del Triunfo
	Magali Maita	Hospital Nacional Hipólito Unanue	Lima
	Juan Carlos Salas	Hospital Regional Docente de Trujillo	Trujillo
	Jose Ormeño	Clínica Good Hope	Lima
