# Supplementary material for: Current clinical nutrition practices in critically ill patients in Latin America: a multinational observational study
Source: Crit Care. 2017 Aug 25;21:227. doi: 10.1186/s13054-017-1805-z (PMC6389103; doi:10.1186/s13054-017-1805-z)
Supplement: Supplementary file 2 — Ethics committee approvals. (PDF 225 kb) [file 13054_2017_1805_MOESM2_ESM.pdf]

Current Clinical Nutrition Practices in Critically Ill Patients in Latin America: A Multinational Observational Study

Ethics Committees

Country	Site #	Site/Ethics Committee	Date	Ref approval
Argentina	5	Hospital El Cruce	9/8/2015	NA
	6	Hospital San Martín	9/28/2015	NA
	7	Sanatorio Allende Nueva Córdoba	7/16/2015	NA
	9	Hospital Cullen	7/7/2015	NA
	10	H. Centro de Salud	8/25/2015	NA
	11	Hospital Británico	8/28/2015	CRIHB #613
	18	Hospital Penna	10/20/2015	NA
	21	Hospital Fernandez	10/29/2015	DI-2015-841-HGAJAF
Brazil	0	CONEP	2/22/2016	1.416.171
	3	Hospital das Clinicas Minas Gerais	3/22/2016	1.461.820
	7	Hospital de Caridade São Vicente de Paulo	6/1/2016	1.570.300
	11	Hospital Felicio Rocho	6/7/2016	1.578.297
	12	Hospital Beneficência Portuguesa De SP	6/9/2016	1.581.880
	17	Hospital das Clinicas São Paulo	7/12/2016	1.633.069
	25b	Unimed Vitoria	7/12/2016	1.633.068
	32	Hospital Sirio Libanes	5/21/2016	1.541.947
	36	Instituto De Assistência Mèdica Ao Servidor Público Estadual – IAMSPE	5/11/2016	1.539.771
	38	Antonio Pedro (HUAP	7/5/2016	1.625.806
	39	Quinta D'Or	7/21/2016	1.644.290
	41	Santa Casa De Misericórdia da Bahia (Hospital St. Izabel)	6/28/2016	1.615.559
	50	Hospital Sta Marcelina de Itaquera	8/3/2016	1.661.786
Chile	1	Dipreca	5/19/2015	NA
	2	Luis Tisne	9/11/2015	1779
	4	Talca	11/3/2015	94
	6	Salvador	8/4/2015	NA
	10	Clínica Dávila	5/13/2015	NA
	13	Naval	10/5/2015	13/2015
	16	Universidad de Chile	9/2/2015	NA
	18	Grant Benavente	10/22/2015	15-09-64
	28	Mutual de Seguridad	10/19/2015	NA

Colombia	6	Hospital Universitario Clínica San Rafael	9/1/2015	CEI-060-2015
	11	Fundación Valle de Lili	11/23/2015	493-2015
	17	Fundación Hospitalaria San Vicente de Paul	8/3/2015	NA
	20	Promotora Clínica Las Américas	8/3/2015	NA
	24	IMAT	8/20/2015	ONC-CEI-CEI-082-2015
Ecuador	1	Hospital Eugenio Espejo	10/20/2015	NA
	2	Hospital de los Valles	10/7/2015	2015-137E
	5	Hospital de la Policia Nacional	7/30/2015	2015-018-DB-HQ1-PN
	6	Hospital Luis Vernaza	8/21/2015	HLV-DOF-CEI-048
	13	Hospital Militar	9/2/2015	015-92-HE-1-10 CBE
Mexico	0	Secretaría de Salud del Distrito Federal	8/6/2015	101-010-14-15
	4	Hospital Civil de Guadalajara	8/20/2015	HCG/CI-1211/15 251/15
	5	Hospital Dalinde	8/26/2015	NA
	6	Hospital General Pachuca	9/15/2015	EI/163
	7	CEMEV (Jalapa, Veracruz)	8/7/2015	29/15
	8	Hospital Regional de Veracruz	8/12/2015	HAEV/DIRECCION/JEIC/DI/091/15 1S.15.1.2
	10	ISEM Centro Médico (Toluca)	8/27/2015	NA
	11	Hospital Central Universitario (Chihuahua)	8/2/2015	CEI-B-139/15
	13	Hospital General de Tijuana	6/1/2015	000672
	14	Hospital Universitario (Monterrey)	8/17/2015	NM15-006
	16	Hospital Metropolitano (Monterrey)	7/30/2015	HMBSSSNL-2015/650
Panama	1	Caja de Seguro Social	9/24/2015	DENADOI-SIBI-01-60-66-15
Peru	1	Hospital Edgardo Rebagliati Martins	8/15/2015	742-GRAR-ESSALUD-2015
	2	Hospital Guillermo Almenara Irigoyen	8/28/2015	195 CEI-OCID-G-RAA-ESSALUD-2015
	3	Hospital Santa Rosa	10/27/2015	6999-2015-OADI-218-HSR-IGSS/DG
	5	Hospital Naval	10/14/2015	050
	6	Hospital Loayza	7/30/2015	1513-HNAL-DG-2015
	7	Hospital Alberto Leopoldo Barton Thompson	10/22/2015	001-2015
	9	Hospital Alberto Sabogal	7/10/2015	512-OCID-RAS-ESSALUD-2015
	12	ESSALUD Trujillo	10/19/2015	144-CIYE-RALL-ESSALUD-15
	15	Hipólito Unanue	10/19/2015	27024
